# Epidemiology of male same-sex behaviour and associated sexual health indicators in low- and middle-income countries: 2003–2007 estimates

**DOI:** 10.1136/sti.2008.030569

**Published:** 2008-07-22

**Authors:** C F Cáceres, K Konda, E R Segura, R Lyerla

**Affiliations:** 1 Cayetano Heredia University School of Public Health, Lima, Peru; 2 UNAIDS, Geneva, Switzerland

## Abstract

**Objectives:**

To conduct a systematic review of published and unpublished data from research and public health information systems on the prevalence of male-to-male sex in the total male population; as well as among men who have sex with men (MSM), data on prevalence of heterosexual activity and heterosexual unions; prevalence of condom use with male and female partners; and prevalence of HIV infection and other sexually transmitted infections (STIs).

**Methods:**

Key indicators were defined (a) among men in the general population: prevalence of sex with a man ever and last year; (b) among MSM: prevalence of heterosexual experiences ever and last year; proportion of male-female transgenders; proportion of sex workers; prevalence of HIV and other STIs, condom use in last sexual encounter; consistent condom use with men last year; never used a condom with a man. With help from key informants, study searches were conducted in Pubmed, LILLACS, institutional databases, conference records and other sources. Methodology and quality of information were assessed, and the best data available for 2003–7 were selected. Indicator estimates from each study were used to propose regional estimate ranges.

**Results:**

A total of 83 new entries were entered into the database in addition to the previous 561, totalling 644. Of these, 107 showing 2003–7 data were selected. Many new studies came from sub-Saharan Africa, portraying hidden HIV epidemics among MSM. The most frequently reported estimate was HIV infection, with high estimate ranges in most of the regions, except for Middle East and North Africa and Eastern Europe. The next most frequently reported was lifetime frequency of heterosexual sex, showing that roughly 50% of MSM ever had sex with a woman. The small number of newer studies reporting prevalence of “sex with a man in last 12 months” between 2003 and 2007, did not warrant enough new evidence to revise our 2005 size estimates for MSM populations.

**Conclusions:**

A considerable number of new studies with estimates of relevance to understanding sexual behaviour and HIV among MSM were identified, with an encouraging amount of new data coming from sub-Saharan Africa. However, limitations in the quality, utility and comparability of available information persist. At least three measures could be promoted for use in surveillance and academic studies: standardised indicators for MSM studies; standardised operational definitions of, and instructions to describe, variables; and standardised research designs and data gathering strategies. A prerequisite for this all is intense advocacy to ensure a social climate in which research into such matters is prioritised, resources are made available as needed and the human rights of MSM are respected.

The last two years have seen a re-emergence of international interest in the role of men who have sex with men (MSM) in HIV epidemics globally. At least three reviews and editorials published in 2007 have contributed to this analysis and have underscored the importance of refocusing prevention work on these populations in more sensitive ways.^1^^–^3 This most likely results from a combination of reasons including: (a) alarming data from higher income countries where a new wave of infections among MSM is occurring, particularly among those who are younger^2^; (b) increasing numbers of studies from lower-income and middle-income countries, where the higher burden of HIV among MSM is a common finding^3^; and (c) a higher recognition of the role of social exclusion and discrimination in determining lower access to adequate prevention and care and, more generally, increasing vulnerability to HIV among men who regularly or occasionally have sex with other men,^4^ regardless of their self and social identification.

Despite the new information, data on HIV transmission through sex between men, and on the incidence and prevalence of HIV and other sexually transmitted infections (STIs) among MSM, are still poor in most of the developing world, and even in many industrialised countries. This is most evident in sub-Saharan Africa, North Africa, the Middle East, Eastern Europe and the Caribbean. However, even in Asia and Latin America, studies conducted usually lack a clear understanding of these populations and their diverse behaviours, and the use of terms and categories is not consistent, affecting interpretation of findings. In addition, many data are collected that remain underutilised owing to a lack of systematic analysis, particularly of behavioural variables.

Clearly, research and surveillance on male same-sex relationships and HIV face many methodological and social challenges. While the use of the category “MSM” underscores the common aspects of biological males having sex with other biological males, it overshadows their diversity and hampers adequate planning for prevention and care.^5^ In much of the world, most MSM also have sex with women, and the low prevalence of condom use among them in sex with both their male and female partners may lead to an underestimation of transmission from men to their female partners.^6^ Another difficulty stems from the fact that HIV prevalence data are usually estimated from samples of MSM selected from high-risk sexual networks, and therefore should not be used in conjunction with population-based estimates of the numbers of MSM^7^; otherwise, the number of HIV-positive MSM would be overestimated. Similarly, the existence of male-to-female transgender people, particularly in South and South-East Asia and Latin America, implies a more nuanced perspective on male-to-male sex. It also affects the ways in which sex questions are interpreted in surveys, particularly given the much higher risk observed in this group.^7^ Moreover, legal frameworks criminalising same-sex behaviour, homophobia, discrimination and human rights violations pose challenges for research as well as the scaling up of interventions and services towards universal access.^8^

Estimating the size of populations of homosexually active men, as well as their HIV prevalence and other characteristics, is critical for estimation of the HIV burden in countries, and planning for prevention, treatment and care, particularly in the many countries with HIV epidemics concentrated among MSM. To provide a scientific underpinning for an improved assessment of the role of male same-sex sexual behaviour in the HIV epidemic in lower-income and middle-income countries, an initial review commissioned by UNAIDS was conducted in 2004–5, and findings were widely disseminated along with informed dialogue and development of policy briefs. Here we provide an update of that review, which benefits from a larger number of better quality studies. Additionally this review includes indicators not published previously on HIV prevalence and preventive behaviours, and discusses additional methodological issues. A parallel study on legal frameworks, violence, stigma and discrimination will be published separately.

## METHODS

Low-income and middle-income countries were classified into nine regions: Asia (East, South, South-East); Africa (East-South and West-Central); Eastern Europe/Central Asia; Middle-East/North Africa (MENA), Latin America and the Caribbean. With slight adjustments (for example, inclusion of transgender estimates), the Access database previously designed was used to enter data from new studies. This study focused on data from 2003–7, which covered both pre-existing and new entries, and included peer-reviewed papers based on data that previously had only been available from conference abstracts.

### Data collection

Searches were conducted in Pubmed and LILACS, conference databases, as well as outside libraries and databases such as the World Bank and the US Census Bureau. These were complemented by searches using more general search engines (for example, Google Scholar). Key reference people also helped locate national AIDS programme official reports and unpublished manuscripts. Studies were included if they showed information on at least one indicator found in the database. Data were entered centrally in Lima, except for some of the data for China (entered locally there). Availability of the full reference was key to assessing the methodology and quality of information in each report.

To systematise estimates of the proportion of MSM in the general male population, the following indicators were sought: “ever sex with a man”, “ever anal sex with a man”, “ever anal and/or oral sex with a man”, as well as the same three indicators within specific timeframes (for example, last year, last 6 months). Similarly, to estimate the relevant characteristics of MSM in published studies, the following characteristics were sought: prevalence of HIV infection (for all MSM and also for male-to-female transgenders); HIV incidence; prevalence of other STIs, lifetime and timeframe specific experience of sex with women; prevalence of condom use in last anal sex with a man; prevalence and condom use in last sex with a woman; consistent condom use for anal sex with a man in specific timeframes; proportion of men who never used a condom with a man; proportion of MSM who are married to or cohabiting with a woman; proportion of MSM who are female/transgender identified; proportion of MSM who are sex workers.

### Data quality assessment

During the first phase of this study, a five-point scale (that is, from excellent to poor) had been designed to assess the quality of each reference based on how informative it was to this analysis, a procedure followed in other databases with quality estimations.^7^ In this phase of the study, criteria for excellence were slightly stricter to reflect the higher number of formal publications. Nearly all reports selected for this update were accessed as peer-reviewed publications and met our criteria for excellence, except for regions where suboptimal data were the only available (for example, conference abstracts in the MENA region).

### Data analysis

Summary tables were prepared to illustrate only the indicators most commonly used to estimate the size and characteristics of MSM populations per region—that is, indicators for which estimate ranges could be shown for more than one or two regions. These estimate ranges were, in principle, limited to data published during the last five years (that is, 2003–7). Data were not pooled within countries, given the extreme diversity of study designs and sampling schemes.

Specific issues were discussed—for example, the conceptual underpinnings of sexuality research, the methodological limitations of the studies reviewed, and also diversity of risk levels among MSM, which makes the estimation of the number of MSM living with HIV uncertain. Finally, a set of recommendations were made on the present use of these estimates, and to improve the availability and quality of future research and surveillance data.

## FINDINGS

### Studies available

A total of 83 new entries were entered into the database that, in addition to the pre-existing 561, made a total of 644. Of these we selected the 107 showing data from 2003 to 2007, which included 14 studies from Africa, 62 from Asia, one from the Caribbean, five from Eastern Europe/Central Asia, 24 from Latin America and one from the MENA region. Compared to the data availability in 2004–5, this search reveals a substantial increase for Africa, and shows the continuation of a very active production of data in Asia and, to a lesser extent, in Latin America. By contrast, data from Eastern Europe/Central Asia were less available, and data from the Caribbean and the MENA region were virtually absent. About 45% of these studies used convenience samples, but a trend was observed towards increasing use of quotas, coupons or respondent-driven sampling. Only two of the 107 studies used random sampling techniques.

### Estimations of the frequency of male-male sexual behaviour

Table 1 shows the total number of studies and range of estimates by region for the two types of data on the prevalence of male same-sex sexual activity for which information across regions was available (that is, primarily ever sex with another man, with 14 studies, and also sex with another man last year, with three studies). These data are also primarily from Asia and Latin America, although in general few studies on male same-sex behaviour among the general population have been conducted. Rather, of the studies from South Asia one includes sex workers and another includes truck drivers, and a South American study reports on lower-income unemployed men, all of which are populations that often have higher rates of male-male sex than the general population. The variable “ever anal sex with a man” was looked for, but not found for this period, and the definition of sex used in these studies was unclear.

**Table 1:** Studies providing estimates of male same-sex sexual behaviour, 2003–7*

		MSM prevalence, lifetime No of studies (range of results) (%)		Sex with a man, last year No of studies (range of results) (%)		References
Africa		2 (1–4)		ND		43, 44
East-South		2 (1–4)		ND		
West-Central		ND		ND		
Asia		7 (4–34)		1 (7)		21, 45–50
East		2 (4–19)		ND		
South		5 (8–34)†		1 (7)		
South-East		ND		ND		
Caribbean		ND		ND		
Eastern Europe and Central Asia		1 (3)		ND		51
Latin America		4 (3–15)		2 (1–14)‡		52–55
Middle East and North Africa		ND		ND		
Total		14 (1–34)		3 (1–14)		

ND, no data.

*Some studies used men attending STI clinics as the study sample.

†The higher end of this range is from two studies—one in India where 23% of the study population had sold sex; the other one among truck drivers in Bangladesh.

‡One paper shows data for male sex contact within last 6 months.

### Frequency of heterosexual activity and heterosexual union among MSM

Table 2 shows the number of studies and the range of estimates for the prevalence of heterosexual sex among MSM (lifetime and for the previous 12 months), and the proportion of MSM married or united to a woman.

**Table 2:** Studies providing estimates of heterosexual activity and of the proportion of transgenders and sex workers among MSM, 2003–7*

		Heterosexual sex among MSM, lifetime No of studies (range of results) (%)		Heterosexual sex among MSM, last year No of studies (range of results) (%)		Proportion of MSM who are married No of studies (range of results) (%)		Transgender among MSM No of studies (range of results) (%)		Sex workers among MSM No of studies (range of results) (%)		References
Africa		3 (41–86)		2 (50–69)		2 (8–15)		1 (2)		2 (74–76)		37–39, 44, 56–59
East-South		1 (41)		2 (50–69)		ND		ND		2 (74–76)		
West-Central		2 (46–86)		ND		2 (8–15)		1 (2)		ND		
Asia		9 (25–73)		10 (11–98)		12 (3–42)		3 (2–63)		6 (12–64)		13, 15, 17, 18, 21, 22, 33, 40, 46, 60–69
East		5 (50–73)		2 (11–52)		7 (7–29)‡		ND		1 (17)		
South		1 (68)		2 (20–98)		3 (21–42)		2 (10–63)		2 (23–64)		
South-East		3 (25–61)†		4 (40–70)		2 (3–13)		1 (2)		3 (12–58)		
Caribbean		1 (78)		ND		1 (41)		ND		1 (45)		70
Eastern Europe and Central Asia		2 (44–53)		ND		1 (7)		ND		2 (5%–15)		31, 32, 71
Latin America		5 (25–64)¶		4 (8–30)§		1 (5)**		4 (4.3–19)		6 (10–31)		23–29, 34, 35, 72, 73
Middle East and North Africa		ND		ND		1 (16)		ND		ND		74
Total		20 (25–86)		16 (8–98)		16 (3–42)		7 (2–63)		13 (5–76)		

ND, no data.

*Some studies include data on male commercial sex workers and/or transgender populations such as *hijras.*

†Lower range is from Bangkok, Thailand.

‡Lower range is from Beijing, China.

¶Highest rate comes from El Salvador and the remaining rates are equal to or lower than 31.7%. One report from each of five Central American countries.

§Includes one study from Peru showing a prevalence of heterosexual sex of 29.6% among all MSM but limited to the last 3 months.

**All data come from one paper showing pooled data from five countries—El Salvador, Guatemala, Honduras, Nicaragua and Panama. Value per country ranges from 1.2%–10.1%.

### MSM subpopulations within study samples

Table 2 also shows the proportion of MSM included in specific studies who are (a) transgender and (b) sex workers. The prevalence of both transgender MSM and sex workers among MSM is almost certainly more an artefactual statement regarding the study and sampling designs in these studies than an accurate portrayal of MSM in these regions.

### HIV prevalence and incidence

Table 3 shows the total number of studies reporting data on HIV prevalence among MSM and transgender populations. In both South Asia and South-East Asia the prevalence of HIV was highly variable within each region. This led to a separation of studies by country and in the case of India reporting north and south India separately. For HIV rates reported from Latin America, a wide variation is also observed both in prevalence figures and in the number of studies by country, so that estimates were also presented by country. The prevalence of HIV among transgender MSM is higher than the estimates of HIV among MSM, evidence of the increased risk and marginalisation of this group. Only one study reports incidence among high risk MSM in Peru in 2002, at 5.1 cases (95% CI: 3.1 to 8.3) per 100 person-years observed.^9^

**Table 3:** Studies providing estimates of the prevalence of HIV infection and preventive behaviours among MSM, 2003–7*

		Prevalence of HIV in MSM No of studies (range of results) (%)		HIV prevalence among transgender No of studies (range of results) (%)		Used condom last anal sex with a man No of studies (range of results) (%)		Consistent condom use for anal sex with a man, last year No of studies (range of results) (%)		Never used a condom with a man No of studies (range of results) (%)		References
Africa		4 (9–25)		ND		2 (6–47)		1 (12–18)		1 (21)		33, 37, 44, 59
East-South		3 (9–25)		ND		2 (6–47)		1 (18)††		ND		
West-Central		1 (22)		ND		ND		1 (11.6)		1 (21)		
Asia		37 (0–40)		7 (0.2–56)		8 (0–82)		4 (0–40)		3 (56–98)		10, 13–16, 18, 20–22, 40, 46, 61, 63, 68, 69, 75, 76
East		14 (0–6)		ND		2 (32–63)		3 (0–40)		ND		
South		9 (0–18)		5 (0.2–56)		1 (20)		1 (20)		3 (56–98)‡		
Nepal		2 (4–5)		–								
Bangladesh		3 (0)		3 (0.2–1)								
North India		1 (2)		ND								
South India		3 (11–18)		2 (40–56)								
South-east		12 (0–28)		2 (7–22)		3 (30–82)		ND		ND		
Vietnam/Indonesia		8 (0–14)†		ND								
Thailand		3 (17–28)		ND								
Caribbean		1 (11)		ND		1 (77)		1 (54)**		ND		70
Eastern Europe and Central Asia		2 (2–5)		ND		2 (37–58)		ND		ND		30, 31, 71
Latin America		10 (8–51)¶		1 (32)		2 (47–54 stable; 56–61 casual partner)		1 (64)**		1 (24)		24, 25, 29, 34–36, 72–74, 77–81
Peru		3 (10–22)		1 (32)		1 (stable partner 54% casual partner 56)		ND		ND		29, 35, 72
Argentina		5 (9–51)		ND		ND		ND		ND		36, 77–80
Bolivia		1 (21)		ND		ND		ND		ND		80
Colombia		1 (20)		ND		ND		ND		ND		80
Uruguay		1 (22)		ND		ND		ND		ND		80
Paraguay		1 (13)		ND		ND		ND		ND		80
Mexico		1 (15)		ND		ND		1 (64)**		ND		81
Central America§		1 (8–15)		1 (24)		1 (stable partner 47 casual partner 61)§		ND		ND		24, 34
Middle East and North Africa		1 (1.4)		ND		ND		ND		ND		74
Total		59 (0–51)		9 (0.2–56)		13 (0–82.0)		5 (0–40)		5 (21–98)		

ND, no data.

*Some studies include data on male commercial sex workers and/or transgender populations such as *hijras.*

†The lowest prevalences were found in non-urban regions.

‡Highest estimate is among truck drivers in Bangladesh.

¶Highest rates (36.5% and 50.9%) come from studies conducted in El Salvador (as part of HIV sentinel surveillance) and Argentina (convenience sample at NGO serving MSM).

§Countries included El Salvador, Guatemala, Honduras, Nicaragua and Panama.

**In both cases the estimates refers to the last 6 months.

††Last 3 months.

### Preventive behaviours among MSM

Table 3 also shows prevalence estimates of preventive behaviours among MSM (that is, condom use at last anal sex with a man, consistent condom use for anal sex with a man during the last year and never having used a condom with a man).

### Other STIs

Although the majority of studies have focused on HIV infection, other STIs have also been studied among MSM populations. The prevalence of syphilis in one study in Africa (Senegal) was 4.3%. In South Asia three studies report syphilis prevalence (that is, 1.5% in Bangladesh, 2.5% in Nepal and 25% in India), and the higher rate was from a study where 64% of the population were male sex workers.^10^^–^12 In South-East Asia three studies found syphilis prevalence to be between 5.5% and 15.5%,^13^^–^15 while in the nine studies from China syphilis prevalence ranged from 2.6 to 13.5%.^16^^–^22 In Latin America, syphilis prevalence was reported in seven studies and ranged from 5.0% to 29%, with highest figures for Argentina (17%) and Peru (29%).^23^^–^29 From Eastern Europe and Central Asia, three studies from Moldova, Croatia and Russia reported prevalences between 10% and 11%.^30^^–^32 No syphilis prevalence data were found from the Caribbean or from the Middle East and North Africa.

Chlamydia and gonorrhoea were primarily tested within the same studies. In the one African study prevalences of chlamydia and gonorrhoea were 4.1% and 5.4%, respectively.^33^ The only study in South Asia was conducted in Nepal with 2.1% of chlamydia infection and 1.7% gonorrhoea infection,^12^ and the study in South-East Asia conducted in East Timor found prevalences of 14.9% for chlamydia infection and 16.1% for gonorrhoea infection.^13^ Two studies in China found chlamydia prevalences of 5.5% and 8.0%, and one of those also reports a gonorrhoea prevalence of 2.7%.^19^ ^20^ In Latin America, only two studies were found with chlamydia and gonorrhoea prevalence estimates: one from Honduras, reporting 12% and 9%, respectively^24^ and another from Peru, reporting 2.4% and 0.0%, respectively.^29^ For Eastern Europe and Central Asia, only one study from Croatia (estimating 9% for chlamydia and 13% for gonorrhoea)^31^ and another from Russia (estimating 5% and 2.5%, respectively)^32^ were found. No data from other regions was identified.

Herpes simplex virus (HSV) type 2 infection was reported in three studies in Asia: China 7.8%,^20^ Nepal 11.8%^12^ and East Timor 29.1%^13^; seven studies in Latin America (Guatemala, Honduras, El Salvador, Nicaragua, Panama and Peru, with prevalences ranging between 43% and 72%^29^ ^34^ ^35^); and one study from Eastern Europe (Croatia, 9.4%^31^). No studies were found from other regions.

Hepatitis B infection was tested for in five studies in Asia, including four in China (with prevalences ranging from 7.5% to 10.3%^16^ ^20^ and one in Vietnam (reporting a prevalence of 31.0%^14^; similarly, from two studies in Argentina (with figures between 22% and 38%^28^ ^36^) and one study from Croatia (with a prevalence of 17.9%^31^). No studies from other regions were found.

### Knowledge and beliefs

An analysis of knowledge and beliefs regarding sexual practices and risk among MSM was beyond the scope of this review. However, some studies also reported on beliefs about anal sex and HIV risk among MSM. In one study from Africa 55% of MSM believed that prevention messages concerning vaginal sex did not apply to anal sex,^37^ and in another study 73% of MSM thought that anal sex was safer than vaginal sex.^38^ These beliefs were evidenced in a third study where participants reported higher condom use for vaginal than for anal sex and frequently blamed their female partners for giving them an STI.^39^ In South Asia, a study among transgender people found that only 14.2% perceived any risk of HIV infection despite practising receptive anal sex and 64% of the sample were sex workers.^40^ In South-East Asia one study showed that 33% of MSM thought that vaginal sex was safer than anal sex.^14^

## DISCUSSION

The re-emerging focus on men who have sex with men as a key component in the struggle to reach universal access to HIV prevention and care^2^ constitutes a sound development in global health policy. Overwhelming evidence about the generally high HIV burden among MSM populations across the globe is available, as the eloquent meta-analysis of HIV prevalence data by Baral *et al* has shown.^3^ However, the challenge emerges of ensuring that such increased interest and resources in fact lead to renewed interventions focused on the prevention, treatment and care for HIV/AIDS that can reach most MSM in most countries, according to their needs and in sensitive, effective and sustainable ways.

An improved planning of prevention, treatment and care services in lower-income and middle-income countries requires solid evidence on various grounds. First, the numbers of MSM in each country should be estimated. Second, the frequency of specific practices associated with varying transmission probabilities should be assessed to estimate risk (for example, anal sex, consistent condom use). Third, the degree of participation of these men in heterosexual sex and unions would improve understanding of the sexual networks in their communities and, especially, the ways in which these men can, or cannot, be reached. Fourth, data on the proportion of MSM who are male-female transgender or sex workers, two special populations thought to be at higher risk for HIV, would also help to better interpret the composition of specific study populations, and provide key information for prevention strategies. Fifth, data on HIV/STI prevalence, incidence and trends among MSM would help define the type and size of specific epidemics and select appropriate interventions and monitoring schemes.^6^ Sixth, data on HIV-related knowledge, beliefs and practices are also fundamental in defining the content and strategies for prevention. Seventh, data on level of community organising and individual participation in such organisations also show the potential role of community organisations as partners and implementers of prevention and care activities. Last but not least, data on legal frameworks and the occurrence of hate crimes and discrimination are crucial to conduct structural interventions in the form of advocacy with governments and international organisations to ensure that any of the above will be possible.

This review sought to identify and summarise some of those types of data. As a positive development since our previous review, the number of studies focused on MSM and HIV has increased in sub-Saharan Africa, where until recently virtually no information was available. Those new estimates are confirming that there are identifiable populations of MSM in sub-Saharan Africa, and that the HIV burden among them is at least as high as among heterosexuals. As van Griensven has pointed out,^41^ progress in the control of HIV in the general population in that region may allow for a focus on specific populations where HIV has a higher potential to develop, particularly if many MSM may have been exposed only to prevention of heterosexual transmission. To date, no resources have been allocated for programmes focused on MSM in this region.

Conversely, information from the MENA region is as limited as before, reflecting the extremely difficult situation of the human rights of MSM in some countries, and probably the unfeasibility of adequate HIV prevention work oriented to MSM at present. This region is an example of the urgent need to address the social and political context before more meaningful data can even start to be collected that do not put research participants at risk of state violence in the first place.

The majority of studies in the most recent period came from Asia, reflecting the increasing interest of many countries (including China) and many international actors to understand the vulnerability in this population, with some parallel progress in the legal environment. Some studies also came from Latin America, although they were not as comprehensive as expected in a region where the legal contexts are probably the most favourable across lower-income and middle-income countries. Finally, limited information was found from Eastern Europe and Central Asia, recognised as mainly affected by an epidemic among injecting drug users, or from the Caribbean, where stigma around homosexuality is still considered appalling.

Data availability, however, is not sufficient to fulfil evidence needs for policy and programmes. Study limitations described previously^7^ persist, affecting the utility of data to inform projections and larger-scale planning. The available data are demonstrative of the lack of a definition of “sex” stated either in the papers describing studies or in the studies themselves. The fact of whether or not participants understood “sex” as any sexual act or specifically as anal (or anal/oral) penetration remains ambiguous despite its implication for risk attribution. Similarly, condom use is assessed in many different ways (for example, non-consistent timeframes; in general or by partner type; focused on use in last sex), making comparability very limited.

Timeframes are also used with little consistency across studies (for example, last month, last six months or 12 months, lifetime) and not always the choice of a timeframe is the best. Recent (for example, last 12 months) experience may indicate recent exposure and also present risk for HIV transmission or acquisition, resulting in highest utility for policy and programmes. However, lifetime experience of sex with other men is useful to assess lifetime risk factors associated with HIV infection. In consequence, asking both questions (that is, lifetime and recent experience) simultaneously provides information on local variations in male sexual behaviour and also allows for the stratification of exposure in two levels, thus allowing for a more nuanced understanding of risk and vulnerability. Most studies identified as providing estimates of the proportion of MSM among males in the general population only assessed lifetime experience of sex with men, an estimate yielding a broad diversity of risk levels among men. However, HIV prevalence figures among MSM are usually estimated among MSM at highest risk in convenience samples in the context of sentinel surveillance. The sizes of MSM populations at highest risk must probably be assessed through other methodologies—for example, capture-recapture analysis.

Most studies do not even explain how they treated male-female transgenders in their study designs, particularly when asking about “sex with other men”, so that readers are left unclear about researchers’ efforts to deal with this topic. The proportion of transgenders in MSM samples is also problematic, since it does not seem to reflect a population-based proportion, but rather a convenience sampling effect based on higher visibility, higher risk (as with sex workers) or economic need. The emerging development of transgender activism in low-income and middle-income countries is pushing against their conflation within the MSM label. Since these populations are socially different and their risk level is apparently higher compared to other MSM, efforts should be made to provide separate estimates for this population in the future, and to develop special programmes for them. This is especially important for regions where they self-identify and are socially recognised as different from other MSM (that is, Latin America and Asia).

The small number of newer studies reporting prevalence of “sex with a man in last 12 months” between 2003 and 2007, did not warrant sufficient new evidence to revise our previous size estimates for MSM populations.^7^ As last time, the indicator most frequently estimated was HIV prevalence, showing figures over 10% across regions, except for the MENA region and Eastern Europe. Estimates of lifetime heterosexual sex among MSM were also frequent. The studies reviewed here have also provided evidence of trends in infection. Notably, a report from Thailand shows a clear increase in prevalence levels among MSM between 2003 and 2005,^42^ suggesting that the epidemic was still in expansion at that time in this population, despite the fact that Thailand was one of the first countries to respond to the epidemic globally.

### Conclusion

In conclusion, a considerable number of new studies with estimates related to sexual behaviour and HIV among MSM were identified, with an encouraging amount of new data from sub-Saharan Africa. While these data provide an important basis for advocacy and initial work, the many limitations of studies reduce their comparability and utility for planning and for the design of truly innovative, high quality interventions.

At least three measures could improve systematic data collection and enhance the possibility of truly evidence-informed planning of HIV prevention and care services for MSM. First, key indicators should be identified to describe the size of MSM populations as well as their characteristics. Second, operational definitions should be standardised for assessment of sexual practices, including timeframes. Third, public health surveillance and research designs and data gathering strategies should also be standardised, including the role of sex workers and male-female transgender populations. All these should result from a broad discussion involving experts from all regions, and should not replace, but complement, local approaches that work. Consensual agreements should be recommended for systematic use in sentinel surveillance and appropriate academic studies.

Newly available data should inform the development of new programmes and interventions, which may involve a combination of elements of traditional programmes (for example, based on education, STI control, counselling and testing) and, potentially, innovative interventions incorporating technological devices (for example, microbicides, vaccines, post-exposure and pre-exposure prophylaxis); provided that they are proved to be efficacious and potential ethical issues are solved. Community input and participation should be ensured. Key requisites for these developments, including the generation of better data from all regions, will be the allocation of resources for HIV programmes oriented to MSM in all low-income and middle-income countries according to needs identified, and the occurrence of transformations that tackle homophobic violence and ensure the respect for the human rights of gay men, transgenders and other MSM.
